# Acaricidal Efficacy of Jasmine and Lavender Essential Oil or Mustard Fixed Oil against Two-Spotted Spider Mite and Their Impact on Growth and Yield of Eggplants

**DOI:** 10.3390/biology10050410

**Published:** 2021-05-06

**Authors:** Saad Farouk, Ahmad B. Almutairi, Yousef O. Alharbi, Waleed I. Al-Bassam

**Affiliations:** 1 Agricultural Botany Department, Faulty of Agriculture, Mansoura University, Mansoura 35516, Egypt; 2 National Organic Agriculture Center, Ministry of Environment, Water and Agriculture, Unaizah 56467, Saudi Arabia; E27247@mewa.gov.sa (A.B.A.); e22929@mewa.gov.sa (Y.O.A.); b.waleed@mewa.gov.sa (W.I.A.-B.)

**Keywords:** eggplants, essential oil, fixed oil, red mites, yield

## Abstract

**Simple Summary:**

The two-spotted spider mite (TSSM) represents the highly polyphagous pest worldwide in protected and open field condition results in a serious economic yield loss (50-100%) in severe infestation conditions. The ecological crisis attributable to the extra-application of acaricides has been an issue of concern in modern decades. It is former to estimate that nearly 2.5 million tons of pesticides are utilized in agricultural production yearly and the global injury evoked by pesticides achieves $100 billion yearly. It is therefore hoped that natural oils can offer alternative options to synthesis acaricides and contribute to pesticide resistance. Application of essential oils of lavender and jasmine, as well as mustard fixed oil, is inducing the plant resistance to TSSM as well as and improving plant growth and yield of eggplants

**Abstract:**

Eggplant is repeatedly attacked by numerous pests, particularly two-spotted spider mite (TSSM), which considerably decline plant productivity. Synthetic acaricides are frequently applied for controlling TSSM, resulting in environmental pollution. The utilization of rational novel substances which repel or prevent TSSM establishment represents a sustainable eco-friendly to reduce the utilization of agrochemicals. A greenhouse investigation was done for assessing the bio-acaricidal activity of mustard (*Brassica juncea* L.) fixed oil (MFO), jasmine (*Jasminum grandiflorum* L.) essential oil (JEO), or lavender (*Lavandula angustifolia* L.) essential oil (LEO), and their influences on eggplant growth and productivity. The results demonstrated that JEO represents the most acaricidal properties against TSSM followed by MFO and/or LEO compared to control. Spraying with natural oils significantly improved eggplant growth, i.e., plant height, number of leaves, and branches/plant, in addition to the leaf area and relative leaf dry mass of the 3rd–5th upper leaves. The JEO had the strongest positive effect compared with other oils or control. Additionally, Natural oils application significantly increased photosynthetic pigment, chlorophyll _a:b_ ratio, and nitrogen, phosphorus, potassium, ascorbic acid, and phenols. The application of oils increased yield and its quality. In this study, JEO (2.5 mL/l) is shown to be extremely promising for the progress of new eco-friendly acaricides, improving plant growth and increasing eggplant yield.

## 1. Introduction

The two-spotted spider mite (TSSM), *Tetranychusurticae* Koch (Acarina: Tetranychidae) represents a highly cosmopolitan and polyphagous pest worldwide in protected and open field conditions [1]. It predominantly prevails in exhaustive high-yield cropping systems, resulting in a serious economic yield loss (50–100%) in severe infestation conditions [2,3]. TSSM in the motile stage normally sucks the sap from the lower leaves’ epidermis, which invokes yellowing and discoloration [2,4,5]. Moreover, severe TSSM invasion induces secondary infestation by fungi, bacteria, and viruses that frequently cause considerable extra injury; additionally, TSSM occurrences induce an allergic syndrome in greenhouse employees [6]. The control of TSSM is particularly problematic due to their short life cycle, potentially explosive population, and their ability to rapidly develop resistance to more than 80 miticides (acaricides) with a few applications [1,7,8]. Consequently, the extension of the chemical acaricide utilized in TSSM control can comprise the commercialization of agricultural systems and induces harmful impacts to the environment and human health, as well as non-target organisms [9]. In addition, the ecological crisis attributable to the application of acaricides has been an issue of concern in modern decades. It has been estimated that nearly 2.5 million tons of pesticides are utilized in agricultural production yearly, and the global injury evoked by pesticides achieves $100 billion yearly. It is, therefore, hoped that natural oils can offer alternative options to synthesis acaricides and contribute to pesticide resistance [10,11].

Natural-based insecticides have been introduced as prospective choices for arthropod management, since they represent a possible supply of bioactive secondary metabolites that have been perceived by the public as comparatively harmless and cause fewer threats to the environment, with negligible effects on human health [12,13]. Furthermore, natural insecticides typically have a mixture of numerous active molecules that exert diverse modes of action, and hence, are possibly capable of efficiently averting the appearance of resistant pest races [14]. Studies have demonstrated that natural oils (essential and fixed oils) are safe, precise in action, eco-friendly, and potentially appropriate for utilizing within integrated pest management programs all over the world within the organic system [12,15,16]. There have been a few studies on fixed oils (FO) as insecticides, for example, Raghavendra et al. [17], with an in vitro study, evaluated the effect of natural fixed oil against TSSM based on the percentage of mortality and the percentage of reduction in egglaying. They indicated that neem oil at 3% can be used to control TSSM.

Essential oils (EO) have obtained a lot of awareness as practical bioactive products, principally in pesticide terms [15,18,19]. The acaricidal action of EO is largely unknown, owing to the complexity of the bioactive substances [20]. The acaricidal activity of EO in direct contact has been tested and fumigant trials have been conducted [21,22,23]. Some studies have attempted to determine the mode of action of EO and their constituents on arthropods [24,25]. The acaricidal properties of EOs may result from more than one mode of action due to the diversity of terpenes and terpenoids or other secondary metabolites that were neuro-insecticides or were species-dependent regarding efficacy; those that showed synergistic efficacy when used in combination, with an octopaminergic system, can mediate the insecticidal activity [21,26]

Eggplant (*Solanum melongena* L.) is among the top 10 most consumed vegetables worldwide. It is cultivated on over 2 million ha, with a production of about 33 million tons. China represents the world’s top eggplant cultivator, accounting for over half of global acreage, followed by India with about one-quarter of the global production; Indonesia, Egypt, Turkey, and Iraq are the other chief eggplant producing countries [27]. Eggplant is an important source of phenolic, antioxidant, and anti-microbial substances, which offer hepatoprotection and cardio-protection, as well as dietary fiber, vitamins, and ions, especially iron; the nutrients that it supplies to the diets of the poor are principally imperative throughout history when other vegetables were in little provide [28]. As for the effect of natural oils on plant growth and productivity, to our knowledge, there are very few reports, i.e., El-Tanany et al. [12], who revealed that EO might be used as biostimulants, which improved plant growth and yield.

Although the acaricidal properties of many essential or fixed oils have been studied against TSSM, not much work has been done on lavender (*Lavandula angustifolia* L.), jasmine (*Jasminum grandiflorum* L.), and mustard (*Brassica juncea* L.) oil against TSSM; additionally, from the current survey, there are no reports on the effect of natural oils on eggplant development and productivity. Therefore, the current study aimed to evaluate the acaricidal activity of EO or FO against TSSM, and their potential as a bio-rational alternative to control that pest under greenhouse conditions, and tested their impacts on eggplant productivity.

## 2. Materials and Methods

A randomized complete design with three replication of four treatments was done during the 2019/2020 season in a controlled greenhouse of the National Organic Agriculture Center, Unaizah (26.085478 °N 43.9768123 °E), Saudi Arabia (SA). The experimental four treatments consisted of jasmine (*Jasminum grandiflorum* L.) essential oil (JEO, 2.5 mL/L); lavender (*Lavandula angustifolia* L.) essential oil (LEO, 2.5 mL/L), mustard (*Brassica juncea* L.) fixed oil (MFO, 5 mL/L), and water as a control. The selected concentrations of the natural oils were based on the preliminary experiment in the lab. and the greenhouse conditions depended on the acaricidal activity and enhancement of plant dry mass accumulation.

### 2.1. Experimental Layouts and Planting Procedure

Before planting, the greenhouse soil was plugged and then divided into eight ridges, each 7 m long and 70 cm apart. Compost (organic fertilizer) as added at 5 ton/ha. The eggplant seedlings were transplanted 50 cm apart, on 1 September 2019, under a drip irrigation system. All agricultural practices were done following the Ministry of Environment, Water and Agriculture, SA, recommendation. The mineral fertilizers that were used during the experimental period were commercial compound fertilizers (© Neutral, macronutrient with micronutrients, Nabat El-Ardh Company, Riyadh, Saudi Arabia) certified in the organic system in SA (©Neutral 4-12-5; from transplanting up to one month; ©Neutral 7-5-4, throughout the vegetative growth period; and ©Neutral 5-5-14, throughout the flowering and fruiting stage) within the fertigation system weekly at recommendation doses.

Once the TSSM infestation percentage naturally reached 50% (1 December 2019), the plants were divided into four groups after the pre-count of the mobile phases of TSSM. Blocks of plants were separated from each other to prevent plant-touching and mites from moving between blocks, and they were surrounded with cloth barricades. The treatments were sprayed until dripping after adding a surfactant at 1%. Foliar applying was repeated, i.e., the first one at 1day post 50% infestation (DPI) and the second at 10 DPI. The entire plants were carefully enclosed by spraying oils, and care was taken to preserve a distance of approximately 30 cm between the nozzle and the plant shoots.

### 2.2. Sampling Dates and Data Recorded

The TSSM (mobile phases) were counted on the 3rd–5th upper leaves of eggplant in the laboratory using a stereo-binocular microscope, two times one week after each spraying, which recorded the number of motile phases of TSSM, after which the corrected efficacy percentage and reduction percentage were determined. The corrected efficiency percentage was calculated according to Henderson and Tilton’s [29] equations:

$$\mathrm{Corrected}\;\mathrm{efficacy}\;\%=\frac{change\%\;in\;treated\;plants\;\pm \;change\%\;in\;control\;population}{100\;\pm \;change\%\;in\;control\;plant\;population}\times 100$$

where:

$$\mathrm{Change}\;\%\;\mathrm{in}\;\mathrm{control}=\frac{population\;in\;control\;plant\;after\;treatment-population\;in\;control\;plant\;before\;treatment}{population\;in\;control\;plant\;before\;treatment}\times 100$$

$$\mathrm{Change}\;\%\;\mathrm{in}\;\mathrm{treated}\;\mathrm{plants}=\frac{population\;in\;treated\;plant\;before\;treatment\;–population\;in\;treated\;plant\;after\;treatment}{population\;in\;treated\;plant\;before\;treatment}\times 100$$

The reduction% were calculated by the formula:

$$\mathrm{Reduction}\;\%=\frac{pretreatment\;counts-average\;number\;of\;life\;TSSM\;after\;treatment}{pretreatment\;count}\times 100$$

Three weeks after the second spraying, plant samples were collected for determination, growth parameter (plant height, number of branches per plant, number of leaves, and branches/plant, in addition to the leaf area and relative leaf dry mass of the 3rd–5th upper leaves), photosynthetic pigment concentration, ion percentage, as well as phenolic and ascorbic acid concentration.

All harvested fruits from each plot were used for determination of the yield and its components (fruit number/plant, fruit dimension ‘length and diameter in cm,’ and total fruit yield/plant). Representative samples of eggplant fruits were arbitrarily acquired from the treatments at the fourth picking to assess the fruit quality attributes (protein percentage, total soluble phenol, ascorbic acid concentration, total acidity percentage, and total soluble solid content). Additionally, oven-dried (at 70 °C) powdered eggplant fruits were used for the estimation of the ion percentage in the fruit (N, P, and K%).

Leaf photosynthetic pigment concentration was assessed via the technique of Lichtenthaler and Buschmann [30] and the optical density of the pigment solution was recorded by using spectrophotometry; the concentrations were expressed as mg/g leaf FW.

For ion estimation [31], dried plant samples (leaves or fruits) were ground to a fine powder, then mineralized by a mixture of sulfuric and nitric acids. Shoot N, P, and K% were determined in a digestible solution. Nitrogen was determined using the micro-Kjeldahl scheme. The colorimetric technique using a UV/VIS spectrophotometer was applied to assess P; finally, a Flamephotometer estimated K.

Total soluble phenols were determined in the methanolic extract; 0.1 mL methanolic extract was mixed with 2.5 mL Folin–Ciocalteu reagent 10%. The mixtures were neutralized by 10% sodium bicarbonate, and optical density was recorded at 765 nm [32]. In preparation of the methanolic extract, an aliquot of frozen plant materials (leaves or fruits) was homogenized in methanol, after which it was centrifuged at 5000 rpm for 20 min.

Ascorbic acid was estimated using the 2, 6-Dichloroindophenol titrimetric technique following the Association of Official Analytical Chemists [33].

Total Acidity was determined following the protocol presented in AOAC [33]. A total of 10 mL of eggplant juice is put into a 100 ml measuring flask, diluted to a tera mark. For the filtered sample, 20 mL of obtained filtrate was taken and inserted into an Erlenmeyer flask. Then, two drops of phenolphthalein were added to the sample and titrated with 0.1 N NaOH until turning pink. The calculation of the total acid was done by the following formula: Total acid = b/a, where a= amount of NaOH 0.1 N for titration (mL) and b = 10 mL of material. Total soluble solids of eggplant were estimated via a manual refractometer at 20 °C, and results were reported as Brix [34].

The data were statistically analyzed through two-way analysis of variance (ANOVA), at a 95% confidence level, using CoHort Software, 2008 statistical package (CoHort software, 2006; release, New York, NY, USA). The means were compared by Fisher’s least significant test (LSD). The statistical significance was considered as * *p*≤ 0.05, ** *p*≤ 0.01; *** *p*≤ 0.001, and ns—not significant. Additionally, Duncan’s Multiple Range Test (DMRT) at *p* ≤ 0.05 was selected to establish the significance of differences among treatments. The values in the tables are the means± standard error (SE)

## 3. Results and Discussion

### 3.1. TSSM Motile Stages Population per Leaf

Data presented in Table 1 show that the changes in TSSM mobile stages population on eggplant as well as the acaricidal effect of studied natural oils. The mean number of TSSM mobile phases ranged between 84–426 per leaf. Application of JEO, LEO, or MFO significantly decreased the TSSM population relative to the pre-count or untreated plants after 1 and 10 DPI. The most effective treatment in this regard was JEO followed by MFO and LEO compared with control. Additionally, the data prove that double spray treatment markedly decreased TSSM mobile stages population to the least level compared to the single spray treatment. The current outcomes showed that the entire natural oils spraying showed efficiency in the control of TSSM to a different degree; the most effective was JEO, which showed higher efficiency and the greatest reduction rates relative to LEO, MFO, or untreated plants. Spraying JEO provides the highest reduction rate (49.03%) in TSSM and high efficiency (68.50%) compared to the other natural oils and control.

Control of TSSM is notoriously difficult, and overall, pesticides are the primary control method; however, they induce detrimental side impacts, i.e., the loss of non-target biota, the occurrence of pesticide-resistant populations and residue concerns, and an outbreak of secondary pests, pest resurgence, and dermal toxicity to the labors exposed in the field [35]. Unfortunately, TSSM has been resistant to most available pesticides and the loss of acaricidal efficiency as a result of resistant mite populations is the major problem encountered [18]. Therefore, it is necessary to find safer replacements that have the prospective to substitute synthetic pesticides and are appropriate for the control of TSSM [36]. The current results and a few reports demonstrated that essential oil (EO)- and fixed oil (FO)-based acaricides can prove as efficient as predictable chemical insecticides alongside soft-bodied pests, including TSSM [13,15,16,18,23].

The acaricidal effect of EO or FO was due to a number of them being selective and biodegradable, and showing a little deleterious impact on non-target biota. EO contains several bioactive compounds, i.e., terpenoids, that possibly exert a regulatory or inhibiting effect on insect life processes [37,38]. Several terpenoids have been revealed to exert acaricidal action, and some constituents are presently utilized commercially as acaricides or repellents [39]. Miresmailli et al. [20], in their research, estimated the acaricidal action of ordinary and artificial rosemary oils on TSSM. They observed that inactive constituents are essential to attain complete toxicity and that the energetic components may have an antagonistic impact on each other. This impact was because of the collective outcomes of increment mortality and decreased productiveness of adult females. Although a precise EO mode of action is still missing, there is proof that the chemically various ingredients of EOs present a wide range of activities, linked chiefly to arthropod nervous systems and detoxification strategies. There is proof that in TSSM, EO may perhaps interact with distinctive molecular targets, i.e., tyramine and octopamine receptors, the GABA system (modification of ionic channels), and the cholinergic system (reserve of acetylcholine esterase), as well as with diverse enzymes, e.g., Cyt P450 monooxygenase, phosphatases, and glutathione-S-transferase [40,41,42,43,44]. One more anticipated mode of action is an intervention with pheromone creation, therefore influencing behavior and reproduction, and interfering with the juvenile hormones’ assimilation as well asecdysones regulating growth and development [45].

Additionally, either EO or FO might be provided as having a bio-stimulatory impact on the assimilation of secondary metabolites, such as total soluble phenols. This buildup could have resulted from the inhibition of catalase activity, which consecutively provokes PAL gene expression and the accumulation of phenolics [46,47]. Nevertheless, total phenols have long been judged as imperative defense-linked substances whose levels are elevated in several resistant cultivars [48]. Outcomes additionally established that spraying of EO or FO significantly raised the percentage of N, P, and K in plant tissue, which reflects an improvement of plant development and plant resistance to insects. This can be attributed to its function in plant metabolic pathways, i.e., to encourage the progress of thicker outer walls and firmness of the cellular membrane in epidermal cells, therefore avoiding insect assault [49].

### 3.2. Growth Characteristics

Data presented in Table 2 show that foliar spraying of EO or FO significantly increased all growth trails relative to untreated control plants. The greatest values of growth parameters were obtained due to spraying with JEO, which increased plant height, branches, and leaf numbers per plant, as well as the area and relative leaf dry mass of the 3rd–5th leaves by 97%, 46%, 37%, 100%, and 38%, respectively, compared to control plants. The data also indicate that spraying of MFO was more effective than LEO in the improvement of growth trials of eggplant.

There is little information about the effect of natural oils on plant growth improvement [16]. The current outcomes proved that eggplant growth was encouragement by foliar application of either EO or FO. The stimulation effect of natural oils on plant growth may be associated with (1) enhancing the antioxidant ability and sustaining plant homeostasis [50], where the antioxidant capacity of natural oils resulted from its internal antioxidant compounds such as flavonoids and tocopherol; (2) increasing the auxin transport as well as controlling the oxidation of indole-3-acetic acid resulted in the accumulation of auxin needed for cell differentiation and an increase in biomass production [51,52]; (3) inducing the development of root system resulted in increasing the capacity of absorption and transport of water and nutrients which are required in plant establishment [52].

### 3.3. Chlorophyll and Carotenoid Concentrations

Chlorosis is a distinctive sign of TSSM-infested plants resulting from chlorophyll degradation caused by TSSM feeding in combination with necrosis-like cell death. In contrast, chlorophyll and carotenoid significantly improved once the application of EO or FO compared to natural infested plants with the strongest effect obtained by JEO (Table 3). The highest concentration of chlorophyll _a_, chlorophyll _b_, total chlorophyll, and total carotenoids were obtained due to JEO application that increased it by 98%, 204%, 129, and 125%, respectively, relative to control plants.

A decline in photosynthetic pigment concentration represents a prime reaction to TSSM attack as it nourishes photosynthetically mesophyll cells. As the TSSM starts feeding on the lower epidermis of leaves, the mesophyll cells disintegrate and a tiny chlorotic area is observed. The quantity and rate of photosynthetic pigment modification have been shown to depend on TSSM mass and nourishing period [53]. The precise mode of action by which TSSM influence plant metabolic processes is not entirely recorded; however, Heng Moss et al. [54] hypothesized that by nourishing principally on phloem tissues, the TSSM modify the pH either on the leaf blade and thylakoid biomembrane, preventing the production of zeaxanthin, or on the stromal plane wherever restoration of violaxanthin occurred. Moreover, Burd and Elliott [55] demonstrated that TSSM nourishing commonly reduces protein assimilation, creating irreparable photoinhibition in addition to blocking electron transport on the acceptor location of the photosystem II reaction center, evoking further decline in the system. In addition, the reduction in chlorophyll and carotenoid possibly resulted from the declining of pigment assimilation that may be caused by the modification in nutrients or lack of accumulation that drains towards the TSSM or to the influence of ROS on these pigments [56]. Additionally, TSSM feeding destroys chloroplasts by puncturing photosynthetically active cells, which leads to vital plant biochemical modifications [57].

The application of natural oils (EO or FO) increased the photosynthetic pigment concentration of eggplant leaves (Table 3). This increase might be because of motivating chlorophyll and carotenoid assimilation and improving the effectiveness of chloroplasts with a superior prospective for resistance and reduction in the photophosphorylation rate that typically occurs once infected [58]. Natural oils were established to boost K concentration [59], which may boost the chloroplast number/cell, cells per leaf, and accordingly leaf area [60]. Additionally, natural oils enhanced the antioxidant capacity of treated plants that nullified ROS and maintained chlorophyll functions and stability [61]. Finally, EO or FO stimulated the carotenoids assimilation and accumulation (Table 3) that defend chlorophyll from oxidation and lastly amplified chlorophyll concentration as recorded in the existing research. Additionally, the application of natural oil increased the nitrogen content (needed for chlorophyll assimilation) and potassium uptake due to improving and regulating the plant architecture through increasing the primary root length, with a higher lateral and adventitious root number per plant, which will improve the absorption capacity of the root and increase the ion uptake [52,62].

### 3.4. Ions and Some Antioxidants

Application of natural oils (FO or EO) increased the nitrogen percentage significantly relative to control plants, and the highest percentage was obtained for JEO application, compared to the other treatments or control. Regarding K and P, the data in the same table show that both lavender and jasmine EO decreased it, while mustard FO increased them by 36% and 83%, respectively, relative to control (Table 4). Regarding the concentration of phenols and ascorbic acid, the data presented in Table 4 revealed that either FO or EO spraying significantly increased it, and the highest concentrations were obtained by application of jasmine EO relative to untreated control plants, which increased it by 142% and 100%, respectively.

The present outcomes and a few earlier reports designate that spraying of natural oils considerably improved the percentage of N, P, and K in plant tissues relative to untreated plants [52,59,63]. This increment could be due to superior root development and elevated root dry matter accumulation as well as sustaining plasma membranes function through the enhancement of the antioxidant capacity after the application of natural oils [51,59]. The increases in root radicular may cause a wide rhizosphere that increases the ion uptake [49,63]. Accordingly, Chapman et al. [52] established that the transcriptional regulatory network regulates the architecture of the plant root system. Additionally, natural oils application possibly has resulted in superior liberate rates of the proton, organic acids, and natural chelators or to a smaller degree by changing the redox perspective of the soil [64].

The application of both EO and FO increased antioxidant compounds such as ascorbic acid and phenols, which offset the destructive impacts of the free radical oxygen on the plants. The total phenolic concentration in the EO- and FO-treated plants was greater than in control plants. This accretion may be attributable to the induction of phenylamino lyase (PAL) gene expression and assimilation of phenolics as well as due to an increase in nitrogen content that is induced by the accumulation of phenols [65]. Thus far, total phenols have long been believed as imperative defense-associated molecules, whose levels are elevated in numerous resistant varieties [48]. Of the plant antioxidants, ascorbic acid represents the most copious and has various biochemical functions, being a substance for ascorbate peroxidase as well as straight nullifying free radicals, and thus, functions as an anti-herbivory agent [66]. An elevated altitude of internal ascorbic is vital to preserving the antioxidant attitude that defends the plant from environmental stress-induced oxidative injure [66].

### 3.5. Yield and Fruit Quality

Data in Table 5 show that exogenous application of JEO, LEO, and MFO markedly increased yield components of eggplant relative to untreated control plants. The greatest fruit number/plant (16.6), fruit length (16.4 cm), fruit diameter (11.6cm), fruit weight/plant (343.3 g), and fruit yield/plant (5910 g) were recorded within the application of JEO with the increasing percentage of 18, 28, 51, 147, and 74% above control plants. The data also proved that MFO was more effective than LEO in increasing eggplant yield. Regarding fruit, yield/plant application of either JEO or MFO significantly increased it, while spraying with LEO non-significantly increased it compared to control plants (Table 5).

As regards fruit quality, data in Table 6 indicate that the application of natural oils (EO or FO) significantly increased both the percentage of nitrogen and protein in the fruit compared to control plants. The highest percentage of N (3.45%) and protein (21.59%) was obtained within the JEO application. Regarding the phosphorous, the application of MFO or LEO significantly increased its concentration, while JEO decreased it relative to control. On the other hand, the highest percentage of K was recorded with LEO application relative to other oils or control. The application of either EO or FO significantly increased the soluble phenol and ascorbic acid concentration as well as the percentage of TSS, compared with control, and the greatest concentration was obtained with JEO spraying, which increased it by 45, 17, and 36% relative to untreated control plants. The data also proved that the application of EO or FO significantly decreased total acidity. The most effective in this regard was JEO.

Commonly, yield components were increased by natural oil application. These results agreed with the finding of Ibtesam Badawy et al. [67] for EO and Dayeswari et al. [16] for FO; they recorded the encouragement effects on the yield of different crops. The increase in yield by natural oils may be attributable to their function in motivating the physio-biochemical pathways that reflected the improvement of plant development (Table 2) and the subsequent energetic translocation of the photoassimilate to sink in eggplants. Accordingly, El-Tanany et al. [12] established that spraying of EO significantly increased Washington navel orange fruit dimension (length and diameter), fruit weight vitamin C content, and TSS when compared to the control. The main causes of the positive effects of natural oils on crop yield might have resulted from the formation of a slim layer of oils over the fruits and encouraged an alteration of microclimatic of fruit [68,69]. Moreover, the application of EO or FO boosts the percentage of K, which accelerates photosynthetic processes and then biomass production [70]. Additionally, EO or FO spraying reduces ethylene creation, resulting in rising fruit yield per plant [71]. Furthermore, the application of natural oils may increase the yield through improving pollen grain germinability and viability as well as improving pollination processes by increasing pollen tube length [72].

The importance of eggplant as a dietary antioxidant source could be linked to the high content of phenolic acids in the fruit flesh and/or the ascorbic acid [73,74]. Abd Elwahab [69], using Nectarine fruits, suggested that the utilization of Bergamot EO demonstrated supreme outcomes in the preservation of overall quality attributes, showing great promise for acquiring a safe and healthy product, particularly under an organic production system. According to San José et al. [75], the relatively high content of antioxidants, particularly phenolics, was determined in the flesh of eggplant fruit.

As for vitamin C, the results indicated that foliar spray with EO or FO significantly increased fruit vitamin C content when compared with the control. Fatemi et al. [76] reported that thyme (*Thymus capitates* L.) and peppermint (*Mentha piperita* L.) oil seriously preserved the quantity of vitamin C and sustained the quality of the *Valencia* orange fruit. Similarly, Zeng et al. [77] and Mohamed and EL-Badawy [78] reported that some essential oils, such as thyme and clove oils, were effective in maintaining the ascorbic acid of orange fruits. Likewise, Abd Elwahab [69] stated that the highest content of vitamin C was obtained from nectarine fruits treated with essential oils compared to control fruits. The author explained that the greatest preservation of vitamin C was recorded with essential oil treatments since these treatments decreased the oxidation in the fruits, as the key substances of oils had antioxidant properties that prevented the oxidation of ascorbic acid.

Concerning the TSS and acidity, it was noted that the application of EO or FO enhanced fruit quality compared to control plants; these outcomes are compatible with those acquired by Rabiei et al. [71], who found that the application of thyme and lavender EO on apple increased TSS as well as decreasing the total acidity and ethylene production. Additionally, Ibtesam Badawy [67], using orange trees, found that earlier harvest spraying with 10% (v/v) lime EO caused a considerable increment in the percentage of TSS compared with control. Moreover, Fatemi et al. [76], using the orange fruit, found that bergamot and peppermint oils showed positive effects on total soluble solids.

## 4. Conclusions

It is concluded that natural oils have great potential to be used for the effective and sustainable management of TSSM in greenhouses within organic farming. Chemical acaricides can be substituted with natural oil, particularly JEO, because of their human safety and eco-friendly properties. Besides the efficiency of natural oils application in TSSM management, it provided some biostimulation on the growth and yield, as well as biochemical attributes of eggplant under greenhouse conditions within organic systems. Yet, extra research is needed to examine the full biochemical and molecular features of natural oil impacts on crop productivity.

## Figures and Tables

**Table 1:** The effect of foliar application with jasmine and lavender essential oils or mustard fixed oil on the eggplant population’s number of TSSM and their efficiency potential and reduction rate.

Treatment	Pretreatment Count	After 1 DPI	After 10 DPI	Efficiency	Reducing Rate
Control	252 ± 3.21 ^b^	390 ± 2.08 ^a^	426 ± 3.21 ^a^	0 ± 0 ^c^	0 ± 0 ^c^
LEO	252 ± 4.72 ^b^	220 ± 5.85 ^b^	186 ± 2.64 ^b^	50.2 ± 1.51 ^b^	19.34 ± 2.80 ^b^
JEO	276 ± 1.52 ^a^	197 ± 2.60 ^c^	84 ± 1.73 ^d^	68.5 ± 0.48 ^a^	49.03 ± 0.41 ^a^
MFO	270 ± 4.58 ^a^	200 ± 4.72 ^c^	100 ± 1.52 ^c^	65.6 ± 1.27 ^a^	44.37 ± 1.96 ^a^
*p*-value	**	***	***	***	***
LSD 5%	12.20	13.42	7.76	4.58	5.62

Lavender essential oil, LEO; Jasmine essential oil, JEO; Mustard fixed oil, MFO; DPI, day post 50% infestation; LSD, Least significance differences; Levels of significance are represented by ** *p* ˂ 0.01 and *** *p* ˂ 0.001. For each parameter, different letters within the column show significant differences between the treatments and control according to Duncan’s test at *p* ˂ 0.05.

**Table 2:** The effect of foliar application with jasmine and lavender essential oils or mustard fixed oil on eggplant growth under TSSM natural infestation in a controlled greenhouse.

Treatment	Plant Height (cm)	Branches No/ Plant	LeavesNumber/Plant	3rd–5th Leaf Area (cm^2^)	Relative Leaf Dry Mass (3rd–5th Leaves, g)
Control	68.6 ± 2.46 ^c^	5.0 ± 0.00 ^c^	47.6 ± 3.48 ^c^	21.21 ± 0.52 ^b^	8.90 ± 0.38 ^c^
LEO	75.4 ± 1.31 ^c^	6.0 ± 0.00 ^b^	55.3 ± 1.76 ^b^	35.86 ± 2.92 ^a^	10.71 ± 0.12 ^b^
JEO	102 ± 1.56 ^a^	7.3 ± 0.33 ^a^	65.3 ± 1.66 ^a^	42.53 ± 3.79 ^a^	12.31 ± 0.14 ^a^
MFO	89.1 ± 4.10 ^b^	7.0 ± 0.00 ^a^	60.3 ± 1.20 ^ab^	38.14 ± 0.52 ^a^	11.51 ± 0.28a ^b^
*p*-value	***	***	**	**	***
LSD 5%	8.49	0.54	7.19	1.71	0.83

Lavender essential oil, LEO; Jasmine essential oil, JEO; Mustard fixed oil, MFO; Least significance differences, LSD; Levels of significance are represented by ** *p* ˂ 0.01 and *** *p* ˂ 0.001. For each parameter, different letters within the column show significant differences between the treatments and control according to Duncan’s test at *p* ˂ 0.05.

**Table 3:** The effect of foliar application with jasmine and lavender essential oils or mustard fixed oil on photosynthetic pigments (mg/g FW) of eggplant growth under TSSM natural infestation in a controlled greenhouse.

Treatment	Chlorophyll_a_	Chlorophyll_b_	Chlorophyll_a:b_	Total Chlorophyll	Total Carotenoids
Control	1.061 ± 0.051 ^b^	0.445 ± 0.10 ^c^	0.417 ± 0.08 ^a^	1.506 ± 0.13 ^b^	0.115 ± 0.01 ^b^
LEO	1.850 ± 0.29 ^a^	1.015 ± 0.03 ^b^	0.590 ± 0.13 ^a^	2.866 ± 0.26 ^a^	0.146 ± 0.005 ^ab^
JEO	2.105 ± 0.249 ^a^	1.354 ± 0.13 ^a^	0.655 ± 0.07 ^a^	3.459 ± 0.31 ^a^	0.259 ± 0.031 ^a^
MFO	1.897 ± 0.083 ^a^	1.023 ± 0.07 ^b^	0.542 ± 0.05 ^a^	2.921 ± 0.08 ^a^	0.186 ± 0.064 ^ab^
*p*-value	*	**	ns	**	ns
LSD 5%	0.65	0.309	0.304	0.723	0.118

Lavender essential oil, LEO; Jasmine essential oil, JEO; Mustard fixed oil, MFO; Least significance differences, LSD; Levels of significance are represented by * *p* ≤ 0.05; ** *p* ˂ 0.01; ns, non-significant. For each parameter, different letters within the column show significant differences between the treatments and control according to Duncan’s test at *p* ˂ 0.05.

**Table 4:** The effect of foliar application with jasmine and lavender essential oils or mustard fixed oil on the ion percentage and concentration of phenols and ascorbic acid of eggplant growth under TSSM natural infestation in a controlled greenhouse.

Treatment	Nitrogen %	Phosphorus %	Potassium %	Phenol (mg/g FW)	Ascorbic Acid (mg/100 g FW)
Control	2.04 ± 0.01 ^c^	0.012 ± 0.0008 ^b^	2.12 ± 0.004 ^c^	0.602 ± 0.02 ^c^	10.0 ± 0.00 ^b^
LEO	2.74 ± 0.16 ^b^	0.012 ± 0.0006 ^b^	1.50 ± 0.001 ^d^	0.961 ± 0.04 ^b^	16.6 ± 1.35 ^a^
JEO	3.19 ± 0.10 ^a^	0.010 ± 0.0003 ^b^	1.95 ± 0.001 ^b^	1.459 ± 0.02 ^a^	20.0 ± 1.15 ^a^
MFO	2.77 ± 0.04 ^b^	0.022 ± 0.0005 ^a^	2.90 ± 0.002 ^a^	1.011 ± 0.02 ^b^	18.0 ± 1.15 ^a^
*p*-value	***	***	***	***	***
LSD 5%	0.325	0.002	0.008	0.096	3.437

Lavender essential oil, LEO; Jasmine essential oil, JEO; Mustard fixed oil, MFO; Least significance differences, LSD; Levels of significance are represented by *** *p* ˂ 0.001. For each parameter, different letters within the column show significant differences between the treatments and control according to Duncan’s test at *p* ˂ 0.05.

**Table 5:** The effect of foliar application with jasmine and lavender essential oils or mustard fixed oil on yield and its components of eggplant growth under TSSM natural infestation in a controlled greenhouse.

Treatment	Fruit No/Plant	Fruit Length	Fruit Diameter	Fruit Weight	Fruit Yield/Plant
Control	13.6 ± 0.33 ^c^	12.8 ± 0.64 ^c^	7.66 ± 0.33 ^c^	138.6 ± 6.35 ^c^	3379 ± 58.8 ^b^
LEO	15.3 ± 0.33 ^b^	14.8 ± 0.30 ^b^	9.66 ± 0.33 ^b^	228.3 ± 21.7 ^b^	4032 ± 165 ^b^
JEO	16.6 ± 0.33 ^a^	16.4 ± 0.21 ^a^	11.6 ± 0.66 ^a^	343.3 ± 19.4 ^a^	5910 ± 532 ^a^
MFO	16.3 ± 0.33 ^ab^	15.1 ± 0.06 ^b^	10.6 ± 0.66 ^ab^	270.6 ± 22.5 ^b^	5103 ± 270 ^a^
*p*-value	***	**	**	***	**
LSD 5%	1.87	1.22	1.41	60.94	1014

Lavender essential oil, LEO; Jasmine essential oil, JEO; Mustard fixed oil, MFO; Least significance differences, LSD; Levels of significance are represented by ** *p* ˂ 0.01 and *** *p* ˂ 0.001. For each parameter, different letters within the column show significant differences between the treatments and control according to Duncan’s test at *p* ˂ 0.05.

**Table 6:** The effect of foliar application with jasmine and lavender essential oils or mustard fixed oil on eggplant fruit quality under TSSM natural infestation in a controlled greenhouse.

Treatment	Nitrogen %	Phosphorus %	Potassium %	Protein %	Phenol (mg/g FW)	Ascorbic Acid (mg/100 g FW)	Total Acidityg Citric Acid/100 g fruit	Total Soluble Solid (Brix)
Control	2.81 ± 0.011 ^d^	0.067 ± 0.001 ^c^	3.95 ± 0.002 ^b^	17.59 ± 0.073 ^d^	2.99 ± 0.02 ^c^	62.0 ± 1.15 ^c^	0.458 ± 0.01 ^a^	4.1 ± 0.10 ^c^
LEO	2.95 ± 0.001 ^b^	0.077 ± 0.001 ^b^	4.17 ± 0.001 ^a^	18.44 ± 0.009 ^b^	3.08 ± 0.03 ^c^	68.6 ± 1.33 ^b^	0.362 ± 0.02 ^b^	4.5 ± 0.18 ^bc^
JEO	3.45 ± 0.001 ^a^	0.042 ± 0.001 ^d^	3.67 ± 0.001 ^c^	21.59 ± 0.010 ^a^	4.27 ± 0.05 ^a^	72.6 ± 0.66 ^a^	0.245 ± 0.01 ^c^	5.6 ± 0.08 ^a^
MFO	2.87 ± 0.008 ^c^	0.095 ± 0.001 ^a^	3.05 ± 0.002 ^d^	17.93 ± 0.055 ^c^	3.38 ± 0.04 ^b^	70.0 ± 1.15 ^ab^	0.256 ± 0.00 ^c^	4.8 ± 0.25 ^b^
*p*-value	***	***	***	***	***	***	***	ns
LSD 5%	0.024	0.005	0.005	0.151	0.141	3.605	0.042	--

Lavender essential oil, LEO; Jasmine essential oil, JEO; Mustard fixed oil, MFO; Least significance differences, LSD; Levels of significance are represented by *** *p* ˂ 0.001; ns, non-significant. For each parameter, different letters within the column show significant differences between the treatments and control according to Duncan’s test at *p* ˂ 0.05.

## Data Availability

The data presented in this study are available on request from the corresponding author.
